# Prioritising methodological research questions for scoping reviews, mapping reviews and evidence and gap maps for health research: a protocol for PROSPECT Delphi study

**DOI:** 10.1136/bmjopen-2024-096298

**Published:** 2025-08-04

**Authors:** Danielle Pollock, Sabira Hasanoff, Grace McBride, Raju Kanukula, Andrea C Tricco, Hanan Khalil, Fiona Campbell, Romy Menghao Jia, Lyndsay Alexander, Micah Peters, Ariany Marques Vieira, Edoardo Aromataris, Jack Nunn, Ashrita Saran, Catrin Evans, Christina Godfrey, Dawid Pieper, Érica Brandao de Moraes, Linda Biesty, Heather Colquhoun, Declan Devane, Elaine Toomey, Barbara Clyne, Ellen Davies, Zachary Munn

**Affiliations:** 1 Health Evidence Synthesis, Recommendations and Impact (HESRI), The University of Adelaide School of Public Health, Adelaide, South Australia, Australia; 2 Knowledge Translation Program, Li Ka Shing Knowledge Institute of St Michael’s Hospital, Toronto, Ontario, Canada; 3 Epidemiology Division, University of Toronto Dalla Lana School of Public Health, Toronto, Ontario, Canada; 4 Queen’s Collaboration for Health Care Quality, JBI Centre of Excellence, Queen’s University School of Nursing, Kingston, Ontario, Canada; 5 Department of Rural and Indigenous Health, La Trobe University, Melbourne, Victoria, Australia; 6 School of Psychology and Public Health, La Trobe University, Melbourne, Victoria, Australia; 7 Population Health Sciences Institute, Newcastle University Faculty of Medical Sciences, Newcastle upon Tyne, UK; 8 The University of Adelaide JBI, Adelaide, South Australia, Australia; 9 Scottish Centre for Evidence-based, Multi-professional Practice: a JBI Centre of Excellence. School of Health Sciences, Robert Gordon University, Aberdeen, UK; 10 Robert Gordon University Scottish Centre for Evidence-Based Multi-Professional Practice, Aberdeen, UK; 11 University of South Australia Rosemary Bryant AO Research Centre, Adelaide, South Australia, Australia; 12 The University of Adelaide Adelaide Nursing School, Adelaide, South Australia, Australia; 13 The Danish Centre of Systematic Reviews, Department of Clinical Medicine, Aalborg University, Aalborg, Denmark; 14 Department of Health, Kinesiology, and Applied Physiology, Concordia University, Montreal, Ontario, Canada; 15 META Group, Montreal Behavioural Medicine Centre, Montreal, Ontario, Canada; 16 Science for All, Melbourne, Victoria, Australia; 17 Cochrane Collaboration Consumer Network, London, UK; 18 Evaluation and Evidence Synthesis, Global Development Network, New Delhi, India; 19 School of Health Sciences, University of Nottingham, Nottingham, UK; 20 The Nottingham Centre for Evidence Based Healthcare, University of Nottingham Faculty of Medicine and Health Sciences, Nottingham, UK; 21 Queen’s Collaboration for Health Care Quality: A JBI Centre of Excellence, Queen’s University, Kingston, Ontario, Canada; 22 Institute for Health Services and Health System Research, Faculty of Health Sciences, Brandenburg Medical School (Theodor Fontane), Rüdersdorf, Germany; 23 Center for Health Services Research, Brandenburg Medical School (Theodor Fontane), Rüdersdorf, Germany; 24 Department of Nursing Fundamentals and Administration, Federal Fluminense University School of Nursing, Niteroi, Brazil; 25 Brazilian Centre for Evidence-based Healthcare Joanna Briggs Institute Centre of Excellence, Sao Paulo, Brazil; 26 University of Galway School of Nursing and Midwifery, Galway, Ireland; 27 Evidence Synthesis Ireland, University of Galway, Galway, Ireland; 28 Cochrane Ireland, University of Galway, Galway, Ireland; 29 Department of Occupational Science & Occupational Therapy, University of Toronto, Toronto, Ontario, Canada; 30 Centre for Health Research Methodology, University of Galway School of Nursing and Midwifery, Galway, Ireland; 31 Insitute for Clinical Trials, University of Galway College of Medicine Nursing and Health Sciences, Galway, Ireland; 32 Department of Public Health and Epidemiology, School of Population Health, RCSI University of Medicine and Health Sciences, Dublin, Ireland; 33 Adelaide Health Simulation, The University of Adelaide Faculty of Health and Medical Sciences, Adelaide, South Australia, Australia

**Keywords:** Research Design, Delphi Technique, Review, Health Education, Health Surveys

## Abstract

**Abstract:**

**Introduction:**

Scoping reviews, mapping reviews and evidence and gap maps (collectively known as ‘big picture reviews’) in health continue to gain popularity within the evidence ecosystem. These big-picture reviews are beneficial for policy-makers, guideline developers and researchers within the field of health for understanding the available evidence, characteristics, concepts and research gaps, which are often needed to support the development of policies, guidelines and practice. However, these reviews often face criticism related to poor and inconsistent methodological conduct and reporting. There is a need to understand which areas of these reviews require further methodological clarification and exploration. The aim of this project is to develop a research agenda for scoping reviews, mapping reviews and evidence and gap maps in health by identifying and prioritising specific research questions related to methodological uncertainties.

**Methods and analysis:**

A modified e-Delphi process will be adopted. Participants (anticipated N=100) will include patients, clinicians, the public, researchers and others invested in creating a strategic research agenda for these reviews. This Delphi will be completed in four consecutive stages, including a survey collecting the methodological uncertainties for each of the big picture reviews, the development of research questions based on that survey and two further surveys and four workshops to prioritise the research questions.

**Ethics and dissemination:**

This study was approved by the University of Adelaide Human Research Ethics Committee (H-2024-188). The results will be communicated through open-access peer-reviewed publications and conferences. Videos and infographics will be developed and placed on the JBI (previously Joanna Briggs Institute) Scoping Review Network webpage.

**STRENGTHS AND LIMITATIONS OF THIS STUDY**

- This study uses a modified e-Delphi process to identify methodological uncertainties about big picture reviews.
- Multiple rounds of online surveys will be conducted to allow for structured and iterative consensus-building and international representation.
- Small group sessions and a final workshop will be held to refine the information from the surveys.
- The development of a strategic research agenda for big picture reviews will allow for the identification of areas which are currently challenging for those conducting these reviews.
- This Delphi is only related to the methodological uncertainties of big-picture reviews in the field of health and the interpretation of findings should be considered within this context only.

## Introduction

 Evidence synthesis methods such as scoping reviews, mapping reviews and evidence and gap maps (EGMs) have been proposed as useful synthesis approaches and are now a popular fixture within the research ecosystem.^1^ These ‘big picture’ reviews (BPRs) respond to research questions and map the available evidence that decision-makers and researchers value.^1^ For example, decision-makers often pose questions that may not be easily addressed through traditional systematic review approaches, such as: ‘What information is there on a particular topic?’ ‘What policy documents exist on this matter?’ ‘What strategies and outcomes are being used?’ ‘What methodological approach is being used within the literature?’ ‘What guidelines already exist on a certain topic?’ Big picture reviews can examine how research is conducted on a certain topic or field, clarifying concepts/definitions, identifying research gaps or linking concepts and characteristics in a field thus improving clarity for those conducting research.^2^ These approaches are valuable not only to researchers and policy-makers, but also to the public, patients and funders, as they allow for the identification of research gaps in an evidence-informed manner, allow for an assessment of the available literature and often provide an interactive online table that can be publicly available. These approaches are an efficient use of limited resources to provide an overview of a field and can help reduce research waste.^3^

Like the continually evolving methods for systematic reviews, BPRs are experiencing growing pains in how they are conducted and their application to the broader evidence ecosystem.^1^ This is understandable and expected as research methods are seldom, if ever, static and are refined and improved with application and practice. BPRs are often criticised for their poor conduct and reporting, and a lack of clarity and consistency in their methodological approaches.^4^ This is a concern, given the exponential growth in the application of BPR approaches across all fields, leading to errors, misjudgement and poor conduct. This can lead to biased conclusions, ill-informed decision-making and poor methodological reputations of reviews. Issues in the conduct and/or reporting of BPRs present several problems, such as contributing to redundant research efforts and research waste.^5^,^8^ Despite the development of conduct guidance and reporting standards (Preferred Reporting Items for Systematic Reviews and Meta-Analyses extension for Scoping Reviews, PRISMA-ScR) for scoping reviews,^9^ there are still several methodological uncertainties and confusion regarding how to plan, conduct, report and disseminate BPRs.^10^

Therefore, there is a need for an internationally developed strategic research agenda that will identify areas within BPRs which require further methodological clarification. This project will develop this research agenda by identifying the methodological uncertainties and providing future research directions for each BPR type.

### Proposed definitions

In this Delphi, scoping reviews, mapping reviews and EGMs will be seen as separate and distinct evidence synthesis types. The definitions for mapping review and EGMs are not well established within the literature and we may find that the definitions of these evidence synthesis approaches are methodological uncertainties themselves.

Scoping reviews, mapping reviews and EGMs may perform similar roles, but their purpose, conduct and reporting requirements differ. Therefore, to ensure that the methodological uncertainties for each BPR are addressed, a top 10 list of methodological research priorities for each of these evidence syntheses will be developed. Working definitions of each BPR are included within table 1.

**Table 1:** Proposed definitions for each big picture review

Scoping reviews	‘Scoping reviews are a type of evidence synthesis that aims to systematically identify and map the breadth of evidence available on a particular topic, field, concept or issue, often irrespective of source (ie, primary research, reviews, non-empirical evidence) within or across contexts. Scoping reviews can clarify key concepts/definitions in the literature and identify key characteristics or factors related to a concept, including those related to methodological research.’^19^
Mapping reviews	‘Mapping reviews are a transparent, rigorous and systematic approach to identifying, describing and cataloguing evidence and evidence gaps in a broader topic area. They aim to answer the question ‘what do we know about a topic,’ or ‘what and where research exists on a particular area."^1^ Booth proposes that ‘A mapping review aims at categorising, classifying and characterising patterns, trends or themes in evidence production or publication*’*.^20^
Evidence and gap maps (EGMs)	EGMs are systematic and visual presentations of the availability of evidence for a particular policy domain. EGMs consolidate what we know and do not know, typically about ‘what works’, by mapping out existing and ongoing systematic reviews and impact evaluations in this field; and by providing a graphical display of areas with strong, weak, or non-existent evidence.’^21^ Mapping can be applied to any research question (eg, prevalence, risk and protective factors, and the consequences of exposure to an adverse event*)*.^22^

In this Delphi study, a ‘methodological uncertainty’ is defined as an aspect of evidence synthesis where there is either limited knowledge or where there are concerns regarding the planning, conduct, reporting and dissemination of BPRs and requirement for clarification and evidence in these areas. A methodological uncertainty can occur when there is no evidence, where there is no consistency in the evidence, or where there is evidence but less articulation of how to apply it in a real-world setting. For example, there may be evidence that is not detailed or specific enough, so the questions the reviewers have can be considered methodological uncertainties. Table 2 presents examples of potential methodological uncertainties that may be identified within this Delphi. There may be methodological uncertainties surrounding the selection criteria, search strategy complexity, data extraction inconsistencies, quality appraisal challenges, the most suitable methods for synthesising data, lack of clarity on reporting standards, ethical implications, software and tool selection, team composition and training, and questions about how and when to update BPRs.

**Table 2:** Examples of methodological uncertainties

Types of methodological uncertainties	Description of methodological uncertainties
Selection criteria ambiguity	Uncertainty about the appropriate criteria for selecting studies or documents for inclusion in a big picture review.
Search strategy complexity	Challenges in designing a search strategy that is comprehensive yet manageable, especially when dealing with diverse types of evidence.
Data extraction inconsistencies	Variability in what data should be extracted from included studies and how to synthesise this data meaningfully.
Quality appraisal challenges	Difficulties in assessing the quality of included studies, particularly when dealing with non-traditional research outputs or grey literature.
Synthesis approaches	Uncertainty regarding the most suitable methods for synthesising data from big picture reviews, especially when the data is highly heterogeneous.
Reporting standards	Lack of clarity on the standards for reporting big picture reviews, which can lead to inconsistent reporting practices.
Ethical considerations	Uncertainty about the ethical implications of conducting and disseminating big picture reviews, particularly when involving sensitive topics or vulnerable populations.
Software and tool selection	Challenges in choosing the appropriate software or tools for managing and analysing data in big picture reviews.
Team composition and training	Uncertainty about the optimal composition of a review team and the level of training required for team members to conduct a big picture review effectively.
Updating reviews	Questions about how and when to update big picture reviews to ensure that they remain current and relevant.

## Objectives

1. To identify and compile the methodological uncertainties for each BPR related to health research.
2. To create a priority list for each evidence synthesis of the top ten methodological research questions that need to be addressed regarding BPRs for health research.

## Methods and analysis

This study will use a modified e-Delphi process and will be reported according to the guidance on Conducting and Reporting Delphi studies.^11^ An e-Delphi will allow for a structured approach in collecting the opinions of the global academic community interested in BPRs and generating a consensus from international experts. A group process such as a Delphi is appropriate as it naturally allows for the academic community to be directly part of the decision-making process in creating a strategic research agenda which could impact (positively or negatively) their work when using a BPR method. The conduct of this e-Delphi has been modelled on the Priority III Study^12^ which aimed to identify the top ten unanswered questions on rapid review methodology.^13^

**Figure 1:**
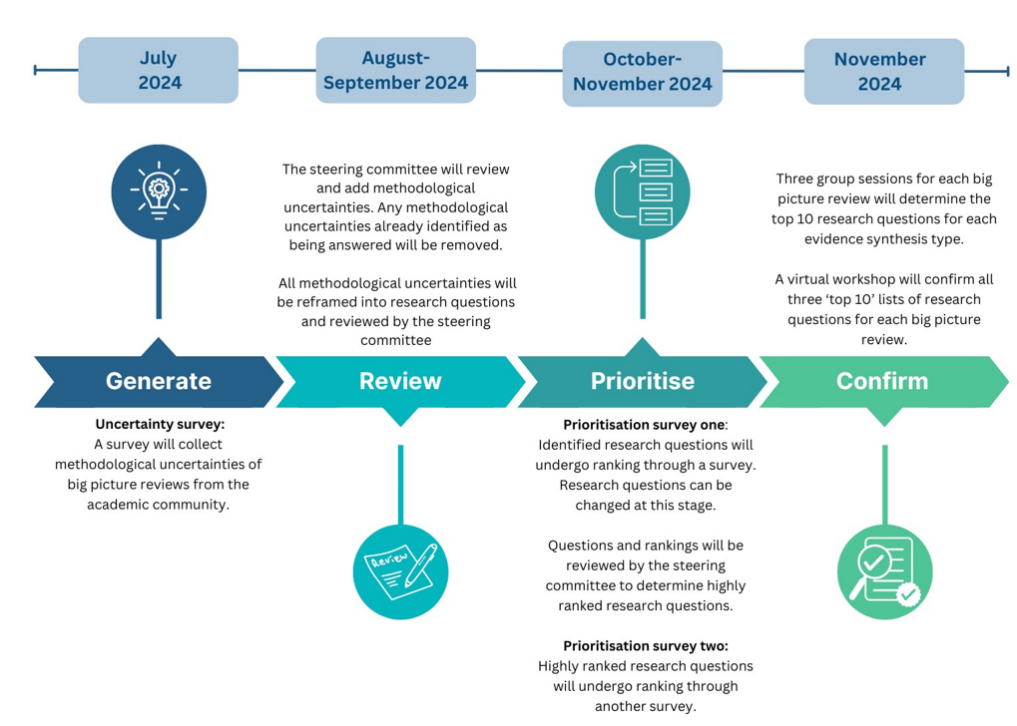
Timeline of the planned e-Delphi process. This figure is a visualisation of the proposed timeline for each step for the current study.

This e-Delphi process will include three online surveys, small group sessions and one final workshop. Figure 1 provides a visual representation of the expected process and timeline that this Delphi study will undertake.

All materials and findings related to this project will be available through Open Science Framework (osf.io/7bkhr).^14^ We will report the method, including public and patient involvement in this project, using ‘Standardised data on initiatives’ (STARDIT)^15^ (https://stardit.wikimedia.org.au/wiki/0202407080037).

### Research steering group

A research steering group has been formed to undertake this research. It will be responsible for providing advice regarding the methodological process of this e-Delphi, assisting in the recruitment of participants, interpreting results, finalising the final top 10 lists for each BPR through workshops, and in the dissemination of the findings. Our steering group involves representatives from the following groups or research projects:

- The JBI Scoping Review Methodology group who have created guidance in the conduct of scoping reviews.
- Investigators who were involved in the development and current update of the PRISMA-ScR.
- The Campbell Collaboration NAVIGATE methodology group on scoping reviews, mapping reviews and EGMs.
- Investigators from the Priority III Delphi, which identified the top 10 priorities for future research for rapid reviews.
- Members of the Evidence Synthesis Taxonomy Initiative (ESTI).

Members of the research steering group have a range of expertise (including a higher degree research student (PhD), researchers (including early career researchers with less than 5 years outside of their PhD completions) within research, methodology and specifically BPRs. We have representation from a patient partner, journal chief editor and healthcare clinicians, which include members from Europe, Canada, India, South America and Australia. While members of the steering group will not participate in the surveys, they will be given the opportunity to contribute to the list of methodological uncertainties during round two (review), and in the final group sessions and workshops.

### Participants involved in online surveys

While these review methods are often used beyond health, due to the limited resources allocated towards this project, we will only be identifying the methodological uncertainties as they relate to health research. We will be attempting to recruit all 11 interest-holders’ groups as defined by the Multi-Stakeholder Engagement consortium.^16^ These include patients, payers of health, payers/purchasers of health services, peer-reviewed journal editors, policy-makers, all members of the research team, producers and commissioners of guidelines, product makers, programme managers, providers and the public. To ensure a broad and diverse understanding of methodological uncertainties, there will be no restrictions on participants’ experience in research, qualifications, previous evidence synthesis experience or familiarity with big-picture reviews in any of the surveys included in this Delphi process (uncertainty survey, prioritisation surveys 1 and 2). We will include anyone interested in improving the evidence that underpins healthcare, including but not limited to members of the public and patients interested in evidence synthesis, researchers, higher degree research students, guideline and policy developers, and journal editors. There will be no limit placed on geographic location of participants, but they must be able to read and provide responses in English.

To recruit participants, the uncertainty survey and prioritisation survey 1 will be advertised through social media channels such as the author teams’ X (previously Twitter) and LinkedIn accounts. The surveys will also be advertised through the JBI Scoping Review Network webpage and its newsletter. Permission to advertise the surveys through appropriate professional newsletters such as the Strategy for Patient Oriented Research Evidence Alliance, Cochrane Collaboration, Evidence Synthesis Ireland, JBI and the Campbell Collaboration will be sought via email. A recruitment email will be sent to the 150+Advisory board members of ESTI. ESTI is currently developing a living evidence synthesis taxonomy and online wiki database.^17^ Those who participate in the first two surveys will be invited to participate in the latter stages of the e-Delphi study through email. To participate in the final prioritisation survey where the questions will be ranked, participants must have participated in the first prioritisation survey.

A social media study promotion kit was developed and approved by the PROSPECT (PRiOritising methodological uncertainties for ScoPing rEviews, mapping reviews, and evidenCe and gap maps in healTh research) steering group.

### Consent

Prior to entering any of the online surveys in this e-Delphi, participants will be provided with information regarding the purpose, the estimated time of completion, benefits and risk of participating, data storage, and withdrawal and complaints procedures. Once this information has been read, participants will be asked to indicate their consent to participate in this process. Participants will not be able to progress to the surveys unless they have confirmed their consent.

To avoid ‘bot’ responses, participants will need to verify that they are not a robot through a captcha verification.

### e-Delphi rounds

There will be approximately four rounds within this e-Delphi to develop the top ten lists of methodological uncertainties for scoping reviews, mapping reviews and EGMs.

#### Round 1: generate

An initial online survey (uncertainty survey) on the Qualtrics platform will be developed to identify methodological uncertainties from participants on BPR methodology. Demographic information such as age group, job title, country, experience and knowledge in evidence synthesis, experience and knowledge in BPRs, and the roles they identify within the community, for example, principal investigator, patient, peer-reviewer (the participant can click as many that are relevant) will be collected.

This project will mostly use questions that have already been developed for the Priority III study^12^ which identified and prioritised the unanswered methodology questions for rapid reviews. These questions were developed with patients and the public, researchers and reviewers, clinicians and policy-makers and were framed with a ‘plan, do and share’ approach to break down the rapid review process. In the initial steering group meeting, it was ascertained that there may need to be specific questions for the current e-Delphi regarding the following:

1. If there are methodological uncertainties regarding the differences between each BPR.
2. The purpose of each BPR.
3. More prompts to assist participants in question 6.

Participants will be able to write any methodological uncertainties they may have regarding each BPR. The uncertainty survey will then be pilot tested by five researchers who are not involved within the steering group to determine the usability of the survey. Table 3 highlights the similarities and differences between the Priority III^12^ and PROSPECT study research questions for the collection of methodological uncertainties.

**Table 3:** Priority III and PROSPECT study methodological uncertainty collection questions

Priority III^12^ —Rapid Review initial survey	PROSPECT—Initial survey
What questions or comments do you have regarding the differences between scoping reviews, mapping reviews and evidence and gap maps?
What questions or comments do you have regarding the purpose of (scoping reviews, mapping reviews and evidence and gap maps)?
(1) What questions or comments do you have about improving the process needed to plan a rapid review successfully?	What questions or comments do you have about improving the process needed to plan a scoping review/ mapping review/ evidence and gap map successfully?
(2) What questions or comments do you have about improving how rapid reviews are carried out?	What questions or comments do you have about improving how scoping reviews/mapping reviews/evidence and gap maps are carried out?
(3) What questions or comments do you have about how the findings of rapid reviews are communicated to people?	What questions or comments do you have about how the findings of the scoping review/ mapping review/ evidence and gap map are communicated to people?
(4) Do you have any other questions or comments on how we plan, do and share the results of rapid reviews?	Do you have any further methodological uncertainties (or unexpected challenges where you did not know how to proceed) related to (scoping review/ mapping review/ evidence and gap map)? Consider the following stages of a review: topic identification; forming a team (including end users); determining objectives/aims/questions; eligibility criteria; searching; extraction; analysis/presentation; critical appraisal; discussion; conclusion/implications and communicating/disseminating findings.

PROSPECT, PRiOritising methodological uncertainties for ScoPing rEviews, mapping reviews, and evidenCe and gap maps in healTh research.

#### Round 2: review

This list of responses generated in round 1 will be reviewed by DPo and SH independently, then will be reviewed together as a team. They will be grouped broadly by similarity of responses, and then checked if there are any uncertainties, which will be determined by members of the steering group. Any comments that are not aligned with the methodology of big-picture reviews will be removed from consideration based on the steering group responses. Responses that have potentially been answered will be flagged by DPo and SH and be further reviewed by the steering group. For a response to be determined as ‘answered’ a published source which has been agreed to by the steering group as adequately describing how to manage this potential methodological uncertainty must be identified. If this occurs, this response will be noted, but removed from any further consideration. A list of these answered responses will be created with the identified sources that respond to them and provided within the supplementary file of the report.

Once determined as a methodological uncertainty, these will be reframed into research questions by DPo, SH and ZM. Once this initial list of research questions has been developed, they will be reviewed by members of the steering group. To assist in the review process, steering group members will assess each question on its relevance to the project (1=no relevance; 2=some relevance; 3=relevant; 4=highly relevant), congruence to the original response collected (1=not congruent; 2=some congruence; 3=congruent 4=highly congruent), clarity (1=not clear; 2=a little clear; 3=clear; 4=very clear), if there are any suggested wording changes (open text) and any further methodological uncertainties (open text) that they believe have not been captured within the survey responses. There must be 80% agreement between steering group members in relevance for the question to progress to the next round. If a question has reached 80% in relevance, however, there are issues regarding congruency or clarity, attempts will be made to reword the question. Those questions will then be examined by the group to assess if the congruency and/or clarity issues have been rectified and can progress to the next round.

#### Round 3: prioritise

Identified research questions will be placed into the first prioritisation survey. Prior to undertaking the questions of this survey, a link embedded within the online survey to a summary of findings of the previous stages will be made available for the participants to review. Once demographic information has been collected, participants will be asked to review the proposed list of questions for each BPR. Participants will be asked to rate each item as either low priority, medium priority or high priority, as seen in table 4. Suggestions for changes to the wording or need for clarification on these questions with an open-ended survey box will be allowed within this survey. These questions, and any suggestions for changes, will then be reviewed by the steering group to determine if any changes should be implemented. Each individual research question will need to have reached at least 80% agreement between groups using weighted voting to proceed to the next round.

**Table 4:** Description of priority levels

Level of priority	Description
Low priority	This research question is not essential and does not need to be prioritised for further methodological clarity.
Medium priority	This research question needs further methodological clarity; however, there is no urgency in providing further methodological clarity.
High priority	This research question is essential and should be prioritised for further methodological clarity.

A second prioritisation survey will then be undertaken to determine final ranking. Prior to undertaking this survey, a link embedded within the online survey to a summary of findings explaining the previous stages will be made available for the participants to review. Participants will be asked to rank their top ten methodological research questions for each review type. No changes to the proposed research questions will be allowed at this stage.

The analysis of these interim priorities in prioritisation survey 1 and 2 will follow the James Lind Alliance approach^18^ where each knowledge user group score will be separated to ensure equality, and that one group does not dominate what the top ten methodological uncertainties are for each BPR. This is important in our Delphi, as we suspect that some knowledge users’ groups may not be as engaged in the process, and we want to mitigate this issue. A scoring system will be implemented based on the number of questions for each prioritisation survey and each group. That is, if there are 10 questions included, then the highest-ranked question will be 10 and the lowest-ranked question will score 1. The scores from each knowledge user group for each question are then added together. The top 10 questions for each BPR will then be reviewed in the virtual workshops in round four. If there are more than ten questions with the same scores, then they will also be presented and discussed in the virtual workshops.

#### Round 4: confirm

This round will include two virtual workshops to determine each review’s initial list of the top 10 research questions based on the findings from round three. Members will be able to discuss any potential stalemates or issues that may occur during round three and decide if these need to be resolved in the final workshop.

Once the first workshop is completed, facilitators (DPo, SH and ZM) will present any conflicting rankings or concerns in the large group to reach consensus. For example, in the situation that there are more than 10 research questions that meet the ranking requirements, the group through the Slido ranking poll will re-rank these options to develop the top ten. Unless there is consensus (through Slido poll) that all these research questions are needed and that more than 10 are required.

Any conflicting rankings or concerns will be presented in this meeting if there is an inability to reach consensus previously. For example, in the situation that there are more than ten research questions that meet the ranking requirements, the group through the Slido ranking poll will re-rank these options to develop the top 10. Unless there is consensus (through Slido poll—over 80% agreement cut-off score) that all these research questions are needed and that more than 10 are required. The facilitators will then present the final questions for each BPR for one last discussion.

## Patient and public involvement

As this is developing the top 10 lists of methodological uncertainties for BPRs, the end-users for this work are mostly pertinent for researchers. It is important, however, to acknowledge the potential impact that the findings of evidence synthesis can have on patients and the public through guideline or policy development. As such, we have included one patient partner in the steering group. The patient involved in the steering group has conducted a PhD in methodology and been a patient representative in several methodological projects. They will be financially reimbursed for their time on this project. Individuals who represent the patient and public experience will also be able to participate in the methodological uncertainty survey and prioritisation surveys 1 and 2.

## Expected outcomes

As the field of evidence synthesis and meta-research continues to grow, so does the need for increased clarity on methodological approaches. BPRs have been criticised for their inconsistent methodological approaches which may be due to a need for more prescriptive guidance. This Delphi will identify the methodological uncertainties presented in the form of research questions that need to be addressed for BPRs to be transparent, rigorous and a useful commodity within the evidence ecosystem. By identifying methodological uncertainties through the development of research questions and consensus, it will better direct and enable methodologists to prioritise the priorities which urgently need addressing. This will allow for better direction and use of scarce resources and hopefully a quicker uptake of the guidance that is developed from these prioritised research questions.

## Expected limitations

Several limitations are anticipated in this study. Given the evolving definitions of these review types, there is a risk that some methodological uncertainties might not be fully captured, necessitating future research to address these gaps. The lack of clarity and clear distinctions between scoping reviews, mapping reviews and EGMs could lead to confusion in participants, affecting the identification and prioritisation of specific methodological uncertainties. Additionally, the study’s focus on the health field may restrict the generalisability of findings to other disciplines. The participant pool is expected to be researcher-heavy, potentially underrepresenting other key knowledge user groups such as policy developers, clinicians, patients and the public, which may result in a bias. There may also be an unequal focus on different review types, with more attention likely given to scoping reviews over mapping reviews or EGMs due to differing levels of review popularity. Additionally, the reliance on online surveys, while efficient, may introduce response biases and exclude non-English speakers, further limiting perspective diversity. Furthermore, identifying if a methodological uncertainty has been responded to or not is dependent on the knowledge of the research steering group, and we may include uncertainties that have indeed been addressed. Acknowledging these limitations provides context for interpreting findings and guiding future research efforts.

## Ethics and dissemination

This study was approved by the University of Adelaide Human Research Ethics Committee: H-2024-188. The results will be submitted for publication in open-access peer-reviewed journals and submitted for presentation at relevant professional conferences, such as the Cochrane Colloquium or the Guideline International Network Conference. Videos and infographics detailing the methods and results of the three lists of methodological uncertainties will be disseminated through social media, such as X (previously Twitter). Professional evidence synthesis bodies, such as Campbell Collaboration and JBI, will be approached to disseminate the findings through their organisation’s social media accounts. The JBI Scoping Review Network website will house all publications, videos and infographics that arise from this work and be promoted through the network’s newsletter.
