# Supplementary material for: Prioritising methodological research questions for scoping reviews, mapping reviews and evidence and gap maps for health research: a protocol for PROSPECT Delphi study
Source: BMJ Open. 2025 Aug 4;15(8):e096298. doi: 10.1136/bmjopen-2024-096298 (PMC12323513; doi:10.1136/bmjopen-2024-096298)
Supplement: Supplementary file 1 [file bmjopen-2024-096298.reviewer_comments.pdf]

PEER REVIEW HISTORY

BMJ Open publishes all reviews undertaken for accepted manuscripts. Reviewers are asked to complete a checklist review form and are provided with free text boxes to elaborate on their assessment. These free text comments are reproduced below.

ARTICLE DETAILS

Title (Provisional)

The PROSPECT Delphi Study: Prioritising methodological research questions for scoping reviews, mapping reviews and evidence and gap maps for health research: a protocol

Authors

Pollock, Danielle; Hasanoff, Sabira; McBride, Grace; Kanukula, Raju; Tricco, Andrea C; Khalil, Hanan; Campbell, Fiona; Jia, Romy Menghao; Alexander, Lyndsay; Peters, Micah; Vieira, Ariany Marques; Aromataris , Edoardo; Nunn, Jack; Saran, Ashrita; Evans, Catrin; Godfrey, Christina; Pieper, Dawid; de Moraes, Érica Brandao; Biesty, Linda; Colquhoun, Heather; Devane, Declan; Toomey, Elaine; Clyne, Barbara; Davies, Ellen; Munn, Z

VERSION 1 - REVIEW

**Reviewer**	**1**
**Name**	**Kaur, Manmeet**
**Affiliation**	**PGIMER, School of Public Health**
**Date**	**15-Mar-2025**
**COI**	**None**

It will contribute towards review articles.

**Reviewer**	**2**
**Name**	**Nunns, Michael**
**Affiliation**	**University of Exeter**
**Date**	**22-May-2025**
**COI**	**None**

This is an interesting and useful project. I found very little to comment on as the protocol is comprehensive and well written.

My only question or discussion point is about the selection of the type of BPRs. Is there opportunity to talk about others such as umbrella reviews or 'mega-' type reviews? Certainly an umbrella review seems more consistently 'Big Picture' than scoping reviews, which could be on quite specific/refined topics (not that i am questioning their inclusion here). Given the planned activity, it feels like a great opportunity to include other review types, so there could be a little more about the justification for the BPRs that have been selected.

VERSION 1 - AUTHOR RESPONSE

Reviewer: 1<br>Dr. Manmeet Kaur, PGIMER<br>Comments to the Author:<br>It will contribute towards review articles.	We thank the reviewer for their positive feedback
Reviewer: 2<br>Dr. Michael Nunns, University of Exeter<br>Comments to the Author:<br>This is an interesting and useful project. I found very little to comment on as the protocol is comprehensive and well written.	We thank the reviewer for their positive feedback
My only question or discussion point is about the selection of the type of BPRs. Is there opportunity to talk about others such as umbrella reviews or 'mega-' type reviews? Certainly an umbrella review seems more consistently 'Big Picture' than scoping reviews, which could be on quite specific/refined topics (not that i am questioning their inclusion here). Given the planned activity, it feels like a great opportunity to include other review types, so there could be a little more about the justification for the BPRs that have been selected.	We appreciate this great question and the thoughtful comments made. We have considered scoping reviews, mapping reviews based on prior categorisation as “Big Picture Reviews” due to their shared purpose and approaches (Campbell F, Tricco AC, Munn Z, Pollock D, Saran A, Sutton A, et al. Mapping reviews, scoping reviews, and evidence and gap maps (EGMs): the same but different— the “Big Picture” review family. Systematic Reviews. 2023; 12(1):45.). We have referenced this paper in the introduction.<br><br>Furthermore, the Evidence Synthesis Taxonomy Initiative (https://evidencesynthesistaxonomy.com/) has also categorised these three types as “Big Picture” for the same reason. While there has been a bit of contention on the use of the term “Big Picture”, there was an >90% agreement rate for the use of this term to describe this group of reviews.
